# Locally Applied Repositioned Hormones for Oral Bone and Periodontal Tissue Engineering: A Narrative Review

**DOI:** 10.3390/polym14142964

**Published:** 2022-07-21

**Authors:** Gamal Abdel Nasser Atia, Hany K. Shalaby, Mehrukh Zehravi, Mohamed Mohamady Ghobashy, Zubair Ahmad, Farhat S. Khan, Abhijit Dey, Md. Habibur Rahman, Sang Woo Joo, Hasi Rani Barai, Simona Cavalu

**Affiliations:** 1 Department of Oral Medicine, Periodontology, and Diagnosis, Faculty of Dentistry, Suez Canal University, Ismailia P.O. Box 41522, Egypt; 2 Department of Oral Medicine, Periodontology and Oral Diagnosis, Faculty of Dentistry, Suez University, Suez P.O. Box 43512, Egypt; 3 Department of Clinical Pharmacy Girls Section, Prince Sattam Bin Abdul Aziz University, Al-Kharj 11942, Saudi Arabia; mahrukh.zehravi@hotmail.com; 4 Radiation Research of Polymer Chemistry Department, National Center for Radiation Research and Technology (NCRRT), Egyptian Atomic Energy Authority, P.O. Box 8029, Cairo 13759, Egypt; mohamed.ghobashy@eaea.org.eg; 5 Unit of Bee Research and Honey Production, Faculty of Science, King Khalid University, P.O. Box 9004, Abha 61413, Saudi Arabia; dzubair@gmail.com; 6 Biology Department, College of Arts and Sciences, Dehran Al-Junub, King Khalid University, P.O. Box 9004, Abha 61413, Saudi Arabia; farhatamu@gmail.com; 7 Department of Life Sciences, Presidency University, Kolkata 700073, India; abhijit.dbs@presiuniv.ac.in; 8 Department of Global Medical Science, Wonju College of Medicine, Yonsei University, Wonju 26426, Korea; pharmacisthabib@gmail.com; 9 School of Mechanical and IT Engineering, Yeungnam University, Gyeongsan 38541, Korea; swjoo@yu.ac.kr; 10 Faculty of Medicine and Pharmacy, University of Oradea, Piata 1 Decembrie 10, 410087 Oradea, Romania

**Keywords:** tissue engineering, periodontium, bone, hormones, drug repositioning

## Abstract

Bone and periodontium are tissues that have a unique capacity to repair from harm. However, replacing or regrowing missing tissues is not always effective, and it becomes more difficult as the defect grows larger. Because of aging and the increased prevalence of debilitating disorders such as diabetes, there is a considerable increase in demand for orthopedic and periodontal surgical operations, and successful techniques for tissue regeneration are still required. Even with significant limitations, such as quantity and the need for a donor area, autogenous bone grafts remain the best solution. Topical administration methods integrate osteoconductive biomaterial and osteoinductive chemicals as hormones as alternative options. This is a promising method for removing the need for autogenous bone transplantation. Furthermore, despite enormous investigation, there is currently no single approach that can reproduce all the physiologic activities of autogenous bone transplants. The localized bioengineering technique uses biomaterials to administer different hormones to capitalize on the host’s regeneration capacity and capability, as well as resemble intrinsic therapy. The current study adds to the comprehension of the principle of hormone redirection and its local administration in both bone and periodontal tissue engineering.

## 1. Introduction

Hormones are unique regulatory chemicals that govern fertility, growth, maturation, and microenvironmental maintenance, in addition to power generation, usage, and storage [1]. Hormones play a fundamental function in the maintenance of the integrity of both periodontium and bone. Many hormones are involved in the maturation, growth, and maintenance of both periodontium and bone, including IGF-1 and thyroid hormone, as well as sexual hormones, calcium-regulating hormones, parathyroid hormone, and vitamin D. Overall, the hormonal effect on periodontal health, bone development, and maximal bone mass is considerable [2,3,4,5,6]. The periodontium is a functionally organized system of several tissues that encircle and reinforce the tooth, in addition to other periodontal tissues, such as periodontal ligament (PDL) and alveolar bone (AB) [7]. Periodontitis is a chronic irritating illness that affects the periodontium. Periodontal disease is described as the deterioration of periodontal tissue, which includes gums, alveolar bone, periodontal ligament, and cementum.

Periodontal diseases have a wide range of pathophysiology.

The interaction between etiological factors and the host’s immune responses results in the creation of many enzymes, chemokines, and mediators, which induces periodontal disease [8].

Bone tissue is made up of many cell types and an extracellular matrix that is mostly made up of collagen proteins. Bone’s primary roles are structural support, mechanical motions, blood cell formation, and tissue preservation; it also serves as a depot of calcium and phosphate ions in the body [9,10]. To maintain skeletal structure, bone resorption and production are closely controlled and managed by bone equilibrium. Osteoblasts, osteoclasts, and osteocytes are all kinds of cells found in bone tissue. Mesenchymal stem cells (MSCs) are responsible for the formation of osteoblasts and osteocytes, while hematopoietic stem cells give rise to osteoclasts.

Osteocytes make about 90% of the bone cell population and serve as the major cells for bone production, mineralization, and cell signaling regulation.

During remodeling, osteoclasts decompose naturally damaged bone and osteoblasts produce new bone, which is then replenished [11]. The rhythm between bone creation driven by osteoblasts and bone degeneration facilitated by osteoclasts is essential for bone homeostasis. Abnormal bone loss occurs when this equilibrium is disrupted, promoting osteoclastic activity, as observed in pathological conditions including periodontitis [12].

Numerous substances have already been discovered as being significant in bone morphology and performance maintenance. Current treatment modalities of both of periodontal and bone diseases, such as, but not confined to, guided tissue regeneration, guided bone regeneration, and surgery, have limited results and can only repair damaged tissues, rather than their regeneration [13].

**Figure 1:**
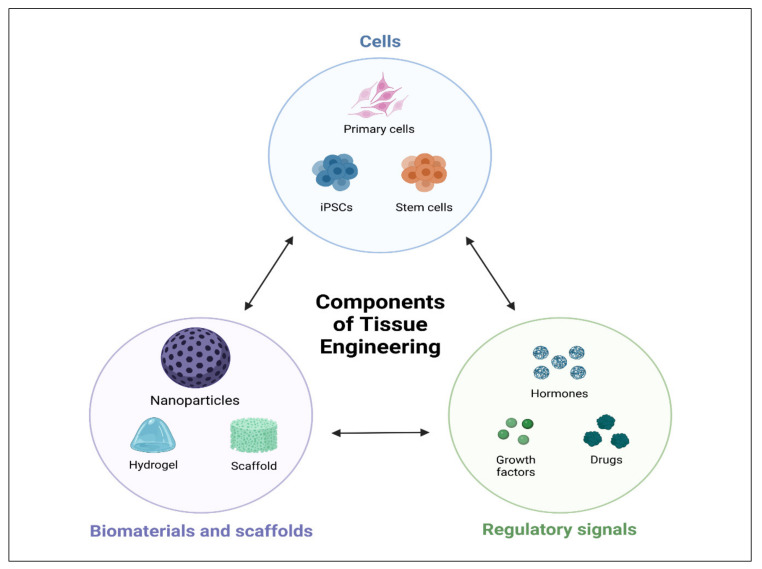
Schematic illustrations of tissue engineering triad. Cell, biomaterials, scaffolds, and regulatory signals.

An innovative alternative is provided by tissue engineering, which is capable of the regeneration of tissues and restoration of their complete function. Tissue engineering is an interdisciplinary approach along with chemistry, pharmaceutics, genetics, and biomedical engineering [14]. Tissue engineering has received attention as a viable strategy in the discipline of tissue regeneration in recent decades, providing a new option for the rehabilitation of teeth, periodontium, bone [15], as well as blood vessels [16,17]. The scaffold, cells, and signaling molecules are three key components of biomedical engineering, as shown in Figure 1 [18]. Several studies have described distinct scaffolds for various types of tissue regeneration; for instance, oral bone and periodontal tissues [19,20]. Stem cells are categorized into totipotent, pluripotent, or multipotent based on their ability to develop into various cell types. [21,22,23,24]. Totipotent cells may give rise to the entire organism, whereas pluripotent cells (iPSC, such as embryonic stem (ES) cells), can actually lead to all cell types in an organism excluding extra-embryonic organs such as the placenta.

Mature stem cells that can develop into a particular cell lineage are known as multipotent stem cells (MSC) [25]. Biologically active substances, such as growth factors [26], medicines [26], and hormones [27], can be delivered locally [28], and were reported to induce oral bone and periodontal regeneration. In this review, we aim to highlight the current strategies and the importance of hormonal repositioning as a viable, economic and safe alternative for growth factors in bone and periodontal tissue engineering, including their opportunities and limitations.

## 2. Properties of Scaffolds for Periodontal and Bone Regeneration

Scaffolds serve as the foundation of tissue-engineered constructions, since they provide dynamical guidance for cells through architectural and biological cues. Scaffolds offer exogenous and/or endogenous cells with geometric support and guidance [29,30]. In general, 3D frameworks with porous structure and interconnections are preferred for anatomical and physiological restoration because the architecture provides an appropriate milieu for cellular contact and scaffold-to-tissue adaptation at the implanted location [31,32]. Given the massive amount of studies, scientific breakthroughs, and technologies, there is frequently a schism between studies and practical implementation, which is commonly known as the “Valley of Death” as a result of the huge amount of enterprises that “die” in between the evolution of innovation and relevant production and marketing [33]. One essential aspect in bridging this gap is the ability to adjust scaffold features in order to meet specific biochemical, clinical, industrial, commercial, and regulatory standards.

**Figure 2:**
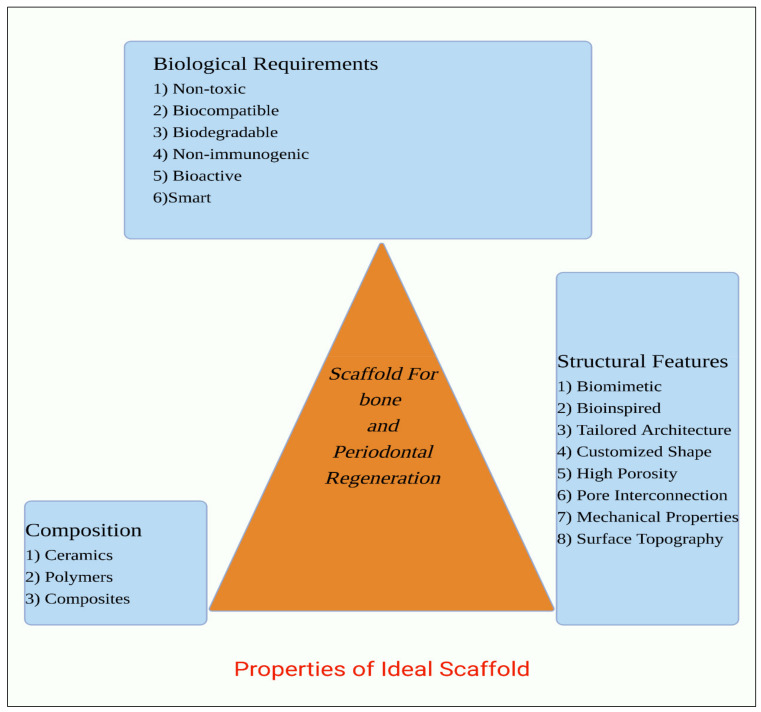
Features of ideal framework for tissue engineering implementations.

An optimal BTE framework should enable or increase cell survival, adhesion, multiplication, and migration, osteogenic differentiation, angiogenesis, and, if needed, mechanical resistance [34]. Furthermore, it should be simple to handle without requiring significant pre-operative procedures and enable minimally invasive insertion. It should be sterilizable using standard procedures and massive-scale replication using economic technologies. Eventually, all of its features must fulfil the standards of the relevant agency or responsible body. The qualities of a scaffold that may be regulated, enhanced, or adjusted to make it acceptable for BTE purposes are classified into three categories: biological needs, structural aspects, and biomaterial composition, as represented in Figure 2.

### 2.1. Biological Requirements

Biocompatibility is the fundamental factor in the implementation of biomaterial frameworks in in situ tissue engineering. The scaffold is biocompatible, produces no immunological rejection, produces harmless breakdown products, and allows cells to attach, develop, proliferate, and grow on the scaffold surface [33,35,36].

### 2.2. Structural Features

Scaffolds must have some porous structure that is required for cell development and motility, nutritional demands, angiogenesis, and spatial arrangement [37]. They ought to have a tailored form to suit the regenerated tissue [38]. They should be thick enough for a prolonged duration to withstand biomechanical pressures until regenerated tissue can bear forces [39]. Another important aspect is morphology, which may be changed by the modification/integration of synthetic ECM and/or biomolecules (hormones, anti-inflammation medications, etc.) to be given in the microenvironment following administration [34].

### 2.3. Biomaterial Composition

They can be injectable or stiff, according to their structure and specific purpose [40,41]. Polymers may be both natural and man-made. Naturally occurring polymers, such as chitosan and collagen, have high biocompatibility, osteo conductivity, and insignificant immune responses [16,42,43]. However, drawbacks include a difficult-to-control deterioration rate and limited mechanical properties.

Synthetic polymers, such as Polylactic acid (PLA), are synthetic materials with a governed biodegradation, the ability to develop or optimize tissue characteristics and construct sophisticated structures, cell adhesion-improved performance, and the capability to release molecules. Furthermore, these polymers can be made at a low cost, in vast homogeneous numbers, and have a long lifespan. One significant disadvantage is that it has a weaker capacity to interact with cells than natural polymers, which have superior bioactive capabilities due to their inherent nature [16].

Hydrogels, polymeric networks that can absorb moisture up to hundreds of times their dry weight, are important forms of polymers used in BTE [44]. This characteristic enables cells to attach, multiply, and differentiate. Natural (chitosan and gelatins) and synthetic (poly(vinyl alcohol)-based) hydrogels can imitate ECM architecture and distribute bioactive compounds [45,46,47]. Gelatin, which is made from the hydrolysis of collagen, is mostly used in the creation of micro particles. Because of their non-toxicity, they are one of the most commonly utilized drug delivery carriers, with storage longevity, cost-effectiveness, and ease of use preparation [48].

Bioactive ceramics (Hydroxyapatite (HA) and bioactive glass might be natural or manufactured. They are chemically comparable to bone and have great compressive strength but low flexibility, offering high rigidity but also fragility [49,50]. Composites are made up of two or more materials with distinct qualities, each with its own set of benefits and drawbacks [50,51,52].

Co-polymeric hydrogels are formed by the combination between different monomers [53], such as PLGA, which is a mixture of poly lactide and polyglycolide and is reported as an ideal contender for BTE implementations, thanks to its biodegradation, and simplicity of production. Polymer composites are mixes of polymeric networks, such as a PLGA-polyphosphazenes blend, that aids in resolving issues caused by PLGA’s harmful breakdown of substances, which can cause tissue inflammation and implant dysfunction, whereas polyphosphazenes do not result in biohazards. As a result, the mix yields degradation products that are almost neutral. Because bone is a composite substance made up of many components of crystalline, HA particles, and organic collagen, polymer-ceramic composites are really biomimetic [52]. They are successful in bone regeneration. In organic inclusions, such as bio ceramic and metal particles, they appear to improve framework mechanical characteristics [54,55]. Table 1 discusses biomaterials’ benefits, drawbacks, and therapeutic applications [56].

## 3. Growth Factors

To offer an osteogenic milieu, GFs are an essential element of periodontal and bone regenerative techniques that regulate essential cellular functions in bone, such as migration, multiplication, development, and matrix synthesis [57] and periodontium [57,58]. Although GFs have been shown to have osteoinductive properties, their clinical value is restricted because of intrinsic features such as limited longevity, short time of action, and rapid processing. As a result, therapeutic doses frequently need large amounts of GFs to establish therapeutic effectiveness. As a result of supraphysiological GF dosages, undesirable effects such as abnormal tissue formation, immunologic response, and cancer risk might arise [59]. All these drawbacks impose searching for viable alternatives for growth factors in periodontal and bone tissue engineering.

## 4. Drug Repurposing

Repurposing a medicine involves using pharmaceuticals that have been licensed for a new indication by regulatory authorities.

An innovative medicine must follow strict criteria to be approved for sale. Because of the varied physicochemical features of chemical entities and the challenge of scaling up manufacturing, identifying a medicine and further developing it requires significant expenditure [60]. This restriction also allows pharmaceutical corporations or academic institutions to use already-approved drugs swiftly and effectively for a novel indication to which patients with that condition do not now have access.

When experimental compounds fail to show effectiveness for a predefined application, repurposing is usually a smart place to start. They can be reintroduced for novel purpose(s), eventually becoming viable medicines, which is especially important in situations of uncommon illnesses, which offer major hurdles in diagnosis, therapy, and limited resources [61,62,63].

Some autoimmune illnesses, infectious diseases, and uncommon malignancies, for example, are not hereditary, making treatment more challenging because they are unpredictable [64]. In comparison with the time-consuming traditional research and development methods, drug repurposing offers a more economical and faster way to bring effective medicines to patients. Furthermore, this technique aids in overcoming the rising costs of drug research, cutting expenditures for consumers and, eventually, lowering the real cost of treatment [65]. Safety and effectiveness information for a novel exploratory molecule are not yet known, leading to higher dropout throughout the drug development process and the most failures in terms of safety or effectiveness [66,67]. In contrary, all toxicology, experimental, and clinical trials effectiveness data for a recycled drug are easily accessible, allowing the investigator to make an educated judgement at each stage of pharmaceutical research [66,67]. The availability of existing information about safety, effectiveness, and the proper delivery route considerably saves research costs and time, resulting in less work being necessary to effectively bring a repurposed medicine to market [45].

The importance and difficulties of medication repurposing are shown in Table 2.

Many pharmaceutical firms are presently using medication repositioning to reconstruct authorized, in addition to previously failed compounds into innovative medicines for a variety of illness conditions, thanks to the enormous promise of a reduced development phase. The current review provides an overview of some of the repositioned hormones and highlight their potential for bone and periodontal tissue engineering.

## 5. Hormones

**Figure 3:**
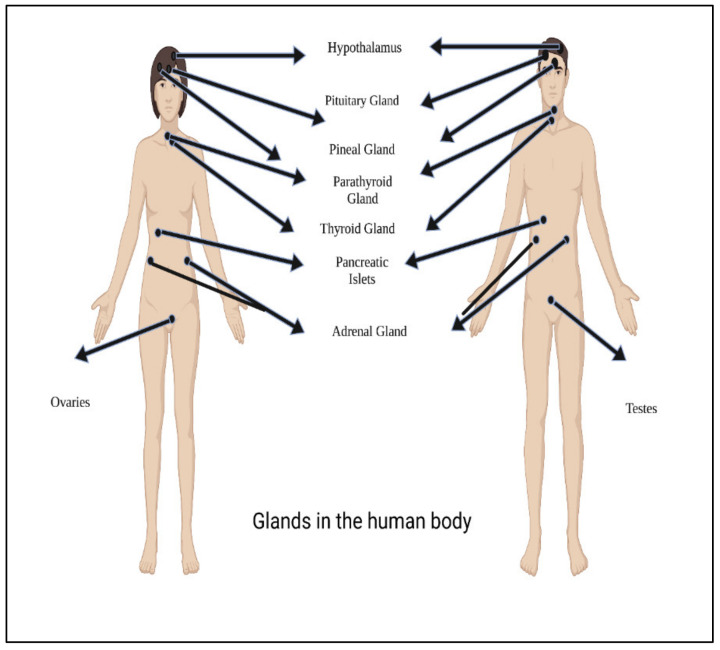
Main glands in the human body.

Hormones are essentially characterized as a stimulants, inhibitors, or chemical messengers that, after being released into the systemic circulation, cause a specific alteration in the cellular activity of target sites. Figure 3 shows main glands in the human body.

**Figure 4:**
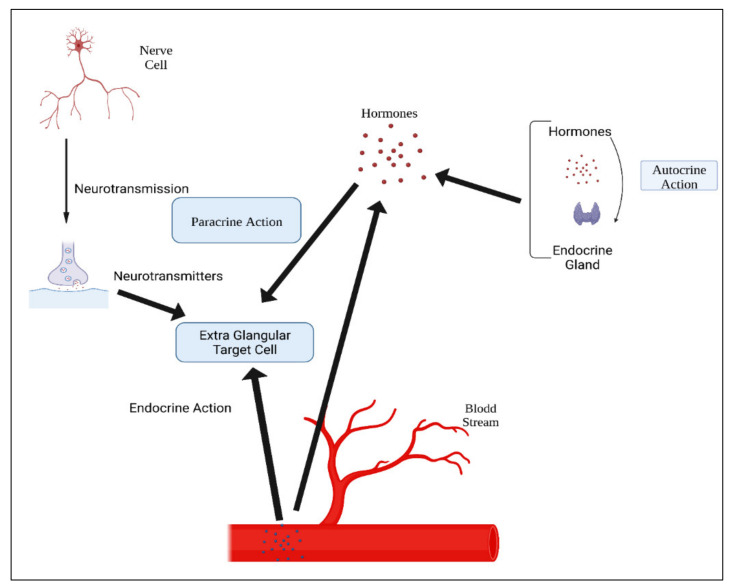
Mechanisms of hormonal actions.

Hormones are classified according to their composition, such as amino acids, tyrosine (catechol amines and thyroid hormones), tryptophan (serotonin), etc., as shown in Table 3. Hormone action could be endocrine (site of their actions distant from the site of release), and may also be paracrine (functioning on nearby cells by diffusion), autocrine (acting on the secreting cells by diffusion), or intracrine (working in secreting cells without release). Agents that work in this manner are frequently referred to as factors instead of hormones, as shown in Figure 4. Indeed, these substances (for example, hormones) may be generated in the majority of cells throughout the body instead of defined endocrine glands [74].

## 6. Examples of Repositioned Hormones for Bone and Periodontal Tissue Engineering

**Figure 5:**
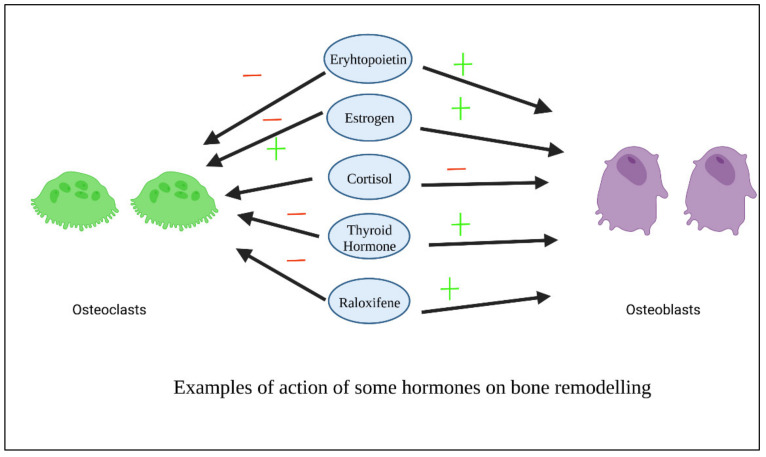
Examples of action of some hormones on osteoblasts.

As previously stated, GFs-based therapies are costly and may cause side effects and immunological reactions in certain individuals. To counteract these disadvantages, various hormones have been designed and tested as viable replacements to growth factors. Hormones are inexpensive to produce, can be readily designed and manufactured, and have little immunogenicity due to their flexibility [75]. Figure 5 shows examples of the action of some hormones on osteoblasts and osteoclasts.

The current research focuses on various hormones locally applied for bone and periodontal tissue engineering, as shown in Table 4.

### 6.1. Thyroxin

Thyroxin is an essential hormone that performs a range of physiological tasks in the human body. One of them is its capacity to stimulate angiogenesis through a variety of methods [88]. By stimulating integrin v3, thyroxin promotes the production of mediators of angiogenesis [89]. Thyroid hormones also influenced cellular metabolic reactions and cell growth [90]. Chitosan/collagen-based thyroxin-loaded hydrogels have a neovascularization capability, which suggests that they might be useful materials for prospective tissue engineering applications [88]. Chitosan composite enclosed with varying doses of thyroxin were demonstrated to be biocompatible, and these hydrogels with pro-angiogenic activities have a high promising applications in periodontal regeneration [76]. In comparison to chitosan, thyroxin-containing membranes demonstrated significant revascularization and rapid wound healing in rats [91].

### 6.2. Oxytocin

Oxytocin (OT) is a fundamental anabolic hormone found in animals during breastfeeding that also has local impacts on bone turnover in addition to the systemic endocrine route [92]. This hormone improves bone production by favorable control of osteoblast development, osteoclast activities, and overexpression of bone morphogenic protein 2 (BMP2) [93,94]. Despite oxytocin being researched in a variety of medicinal applications, its influence on in situ osteogenesis has not been explored, most likely because of its limited half-life and instability versus hydrolysis [95]. The impact of this hormone is only temporary in the absence of an adequate carrier and encapsulation technique, and the physicochemical stabilization cannot be preserved over the bone healing period. Thanks to their unique features, poly (D, Llactide coglycolide) PLGA copolymers have been used as local drug carrier for different types of biomolecules [96]. Sustained release micro spherical oxytocin hormone in a polymeric hydrogel scaffold mixed with biphasic calcium phosphates combination promotes bone repair in the rat calvarias [97]. Furthermore, OT-loaded b-TCP increases osteogenesis in rats with calvarias bone defects via an osteoinductive mechanism of action [77]. In vitro, OT increased PDLSC proliferation, aggregation, and osteogenic differentiation. Additionally, OT’s influence on osteogenic development was driven by the ERK and AKT pathways. As a result, OT has the potential to be used in periodontal regeneration [98].

### 6.3. Dexamethasone

Dexamethasone (DEX) has been demonstrated to enhance osteoblast development in vitro and bone tissue creation in vivo by enhancing osteoblast-related gene transcription [99,100]. DEX has long been employed as an osteoinductive factor due to its excellent integrity as well as osteogenesis [101,102]. High DEX concentrations, on the other hand, would inhibit osteoblast growth and create hazardous adverse effects [101,103], As a result, its additional functional applicability in bone tissue engineering is limited. Thus, prolonged release of DEX is essential to maximize effectiveness while minimizing negative effects on bone regeneration. Porous bio composite matrices comprise the chitosan-alginate-gelatin scaffold in addition to the accumulation of calcium phosphate and DEX-loaded nano silica. Doping was manufactured and demonstrated increased growth and osteogenesis in rats, suggesting that they might be extremely good as potential local insertable frameworks for possible uses in bone tissue engineering [78]. Dexamethasone (DEX) has been demonstrated to initiate bone marrow differentiation as well as guide cells toward maturation [104,105]. Injectable hydrogels loaded with dexamethasone have a promising potential as an injectable drug-depot for bone repair therapy in cases of chronic inflammation [106].

### 6.4. Androgens

In males, testosterone is the major sexual hormone and anabolic factor. In humans, testosterone is crucial in the male sexual organs, for example the testes, as well as in the promotion of secondary sexual traits such as increased muscular and bone mass [107]. PLGA-coated pericardial inserts or membranes combining topical gradual administration of supplementary quantities of testosterone and alendronate may be a viable approach for stimulating in situ osteogenesis, leading to enhanced implant osseo-integration and repair of bone defects and fractures [79]. In mice, testosterone delivered with a scaffold has similar effects to the Bone Morphologic Protein-2 in enhancing bone regeneration [108].

### 6.5. Parathyroid Hormone (PTH)

The endogenous parathyroid hormone is a critical mediator of bone remodeling as well as a crucial regulator of calcium-phosphate equilibrium. This hormone promotes bone formation by activating numerous mechanisms involved in stem/preosteoblast cell osteo differentiation. Inhibiting osteoblast apoptosis can also increase the quantity of osteoblasts. PTH causes osteoblasts to release a number of growth factors, and it causes osteocytes to produce less sclerostin and DKK, two anti-osteoclastic and Wnt signaling inhibitors. Furthermore, PTH may indirectly trigger osteoclasts to accomplish bone resorption. PTH stimulates osteoblast RANKL synthesis and increases RANKL binding to osteoclast surface receptors, resulting in osteoclast activation [109]. The amount and duration of PTH exposure influence bone production (anabolism) and bone resorption (catabolism). Constant and high hormone dosages promote bone breakdown, whereas minimal and inconsistent levels promote osteogenesis and increased mineral density [110].

PTH has been demonstrated to significantly speed up fracture healing [111,112]. As a result, local PTH delivery to bone abnormalities might be a practical solution and alternative to auto graft [113]. Huang et al. have developed a controlled delivery method using a parathyroid hormone derivative (PTHrP-2) and a meso-porous bioactive glass scaffold. In the PTH-loaded scaffold, BMSC responses to this scaffold revealed increased osteogenesis and osteoinduction. Furthermore, the PTHrP-2-loaded scaffold had lower osteoclastogenesis than the unmodified peptide-loaded scaffold [114]. Ning et al. created an injectable Gelatin hydrogel for the delayed release of abaloparatide in a trial. This scaffold resulted in a greater bone formation and mineral density [115].

### 6.6. Insulin

Insulin is a hormone which affects energy production and balance, as well as being an important part in bone formation metabolism. Skeletal anomalies linked to Diabetes type I can be cured with insulin treatment [116,117]. Clinically, it is frequently noted that insulin shortage increases the possibility of fracture. The use of insulin therapy dramatically boosted bone formation in patients with type 2 diabetes, which can minimize the risk of fracture [118,119]. Insulin/IGF-1 has been proven in vivo to induce angiogenesis and give nourishment for bone growth. [120,121,122]. Insulin can successfully enhance local skull bone growth in the mouse skull by raising the quantity of bone forming cells and the surface area of the osteoid [123], and has the ability to control osteoclastic activity [124]. In recent years, research has discovered that IGF-1 can also influence the formation and maturation of osteoblasts, hence increasing bone repair [125]. Given the success of nanoparticles in drug loading, a variety of insulin carriers have been innovated, which could be breakthroughs in bioengineering technology [126]. In another study, insulin-loaded poly lactic-co-glycolic-acid (PLGA) Nano spheres were incorporated into nano hydroxyapatite/collagen (nHAC) scaffolds, where insulin was successfully distributed from the nano spheres and aided bone regeneration in significant size impairments in the rabbit mandible [81]. Furthermore, insulin-encapsulated PLGA microspheres greatly enhanced the insert’s stability in rabbits at Week 4, indicating that it is possible to lower the implant’s early failure rate without affecting serum biochemical markers [127]. New bioactive injectable composites loaded with insulin have been developed and might be used to treat bone defects, notably as an economic promotion/substitute to BMP-2 approaches [128]. Local insulin infiltration at the implant–bone contact has the potential to have significant therapeutic ramifications by spontaneously increasing the effectiveness of oral implantation in diabetic rats [129].

### 6.7. Estrogen

Estrogen is a natural steroidal hormone that regulates bone mass and maintains bone tissue balance. The estrogen’s activity is directly connected to the regulation of osteoblast proliferation and differentiation. In addition, estrogen reduces apoptosis in osteocytes and osteoblasts while inducing apoptosis in osteoclasts. By decreasing the synthesis osteoclastic mediators, estrogen reduces the creation of active osteoclasts. Moreover, it increases the creation of osteoprotegerin by osteoblasts and osteocytes (OPG) [130,131]. 17-estradiol (E2) is the most powerful hormone in the body system, and it adheres to estrogen receptors (ERs) in both bone cells and MSCs. Estradiol can encourage MSCs to differentiate into osteoblasts and improve osteogenesis by boosting the expression of BMP-2, TGF-1, and IGF-1 [132]. Estrogen activity causes bone remodeling to be balanced and bone metabolism to be modulated. As a result, estrogen deprivation reduces osseous density, raises the possibility of osteoporotic fractures, and causes bone loss [133]. Systemic estrogen treatment can help reduce osteoporotic fractures in postmenopausal women. Accumulation in organs, on the other hand, generates negative consequences, for example, cardiovascular disease and breast cancer [134]. A controlled release to administer the lowest therapeutic dosage while avoiding systemic adverse effects may be a desired method for extending estrogen clinical uses. Various tissue-engineering technologies have been investigated in order to create local delivery for an osteoporotic bone fracture. Nano materials have recently been identified as an excellent choice for the transport of biomolecules. 17-estradiol (E2) was put into a nano fibrous matrix, which demonstrated improved cell growth and osteoblast development mediators [84]. Chen et al. recently created a core-shell nano composite for bone-targeted hormone administration, loading E2 in an EDTA- adjusted nano composite. Sustained E2 release resulted in increased ALP, OPN, OCN, and calcium deposition in MC3T3-E1 preosteoblasts. Furthermore, intraperitoneal injection of an E2-loaded nano composite decreased bone deterioration in ovariectomized rats [135]. Morales et al. employed a mixture of 17-estradiol and BMP2 to cure calvarias bone deficiency in rats in another investigation. The injectable hydrogel scaffold is made up of BMP2-loaded PLGA micro particles and 17-estradiol-loaded PLA microspheres. Therapy with BMP-2 coupled with 17-estradiol has a synergistic impact and restored the estrogen shortage in osteoporotic mice, resulting in more bone production enhancement than the BMP2-alone treated group [136].

### 6.8. Selective Estrogen Receptor Modulators (SERMs)

Selective estrogen receptor modulators (SERMs) are non-steroidal compounds that have estrogenic actions on the bone, vascular system, and lipid profile, while also having anti-estrogenic effects on the breast and uterine [137,138]. Through an estrogenic action on the skeletal structure, they promote endochondral ossification, bone production, and callus remodeling [139]. By reducing osteoblast and osteoclast bone turnover, selective estrogen receptor modulators decrease bone degradation and lessen the fracture probability [140,141]. Several SERMs are now being used in clinical settings, including Raloxifene, Tamoxifene, bazedoxifene, Lasofoxifene, Ospemifene, Arzoxifene, Droloxifene, Idoxifene, and Fulvestrant [142,143]. Tamoxifen is a therapy for breast cancer that reduces osteoclast-mediated bone resorption [144,145]. Both raloxifene and bazedoxifene are SERMs that have been demonstrated to reduce bone resorption activity in postmenopausal osteoporosis patients [141,143,146,147] and have been utilized to keep bone fragility fractures at bay. SERM binding to estrogen receptors (ERs) modifies the receptor’s structure or capacity to form a combination with co-regulators, altering their expression levels [148,149,150,151,152].

#### Raloxifene

Raloxifene (RLX) is a second-generation selective estrogen receptor modulator (SERM) that is now approved as an osteoporosis medication. Raloxifene has an estrogen-like action on bone, and has been found to improve bone mass density (BMD) and preserve bone health [153]. In comparison with untreated tibia perforations, poly-lactic-co-glycolic acid (PLGA) loaded with raloxifene hydrochloride accelerated bone growth in non-critical sized lesions in the rats’ tibia [154]. In a recent study [155], in vitro testing was performed using a scaffold loaded with PLGA microspheres containing RLX, with RLX dosages ranging from 0.1 to 10 g. The conclusions demonstrated that the frequency of RAL liberation from the microparticles was slow and regulated, resulting in superior cell survival at all concentrations, considerably increased cell proliferation, greater mineralization capability, and ALP activity. In osteoporotic rabbits, a TiO2 nanotube arrays (TNT)/raloxifene (RLX)/layer-by-layer/alendronate (RLX/LBL-Aln) implant may effectively accelerate the creation of new bone surrounding the implant and improve bone binding [82]. A new nano-fibrillated cellulose/cyclodextrin-derived 3D framework loaded with raloxifene hydrochloride improved cell aggregation and alkaline phosphatas expression, all of which are required for bone mineralization. The findings revealed a unique, risk-free, and advantageous strategy to bone engineering [156]. A thin meso-porous TiO2 carrier matrix combined with both Alendronate (ALN) and Raloxifene (RLX) can be utilized to speed up implant retention in trabecular bone in rats [157].

### 6.9. 1, 25(OH) 2 Vitamin D3

Vitamin D is a fat-soluble hormone that governs bone development and strength and helps to maintain calcium-phosphorus proportions. Scientific proof suggests that vitamin D plays an autocrine function in bone production, mineralization, and degeneration. 1, 25(OH) 2 D3 influences osteoblastic protein production via the (MAPK) ERK1/2 system [158,159]. Many studies have demonstrated that vitamin D has a high capability in both osteoinduction and odontiinduction. At modest doses of this chemical, the expression of OCN, OPN, DSPP, DMP-1, and bone mineralization has enhanced [160]. Bordini et al. created a scaffold loaded with 1 nM 1, 25-dihydroxy vitamin D3. They discovered that vitamin D3 can boost odontoblastic marker expression [161].

A cellulose/hydroxyapatite/mesoporous silica scaffold was created and supplemented with vitamin D3 in a similar work. In vitro research revealed that vitamin D3 might improve cell adhesion and proliferation (MG63). Furthermore, the ALP activity and calcium accumulation assays validated the synergistic effects of hydroxyapatite and vitamin D [162]. Sattary et al. recently created a polycaprolactone/gelatin scaffold incorporating HA nanoparticles. They discovered that including vitamin D into the framework blends increased osteogenic development and hardening potential in hADSCs. On day 14, the synergistic impact of vitamin D and HA nanomaterials resulted in an increase in the osteogenic marker in the PCL/Gel/nHA/Vit D3 scaffold group [85].

### 6.10. Melatonin

Melatonin’s (ML) involvement in hard tissues has gotten a lot of attention [163,164]. The indoleamine ML (N-acetyl-5-metoxy-tryptamine) is produced and released by the pineal gland in a circadian rhythm [165]. Melatonin is also produced in possibly all organs in numbers of orders of magnitude greater than in the pineal gland and bloodstream [166]. ML may be implicated in the formation of hard tissues such as bone and teeth [167]. ML stimulates alkaline phosphatase activity and tissue mineralization [168]. As previously indicated, ML has been employed for its anti-inflammatory, antioxidant, and free-radical-scavenging qualities [169,170] and cytoprotective properties [171,172]. When there is a large quantity of ML, the generation of inflammatory mediators decreases via modulating the NFkB activity, which contributes to the signaling route.

While the favorable benefits of ML on periodontal regeneration have been proven in gingival fibroblasts as well as in experimental animals, more research is needed. [171]. ML has a circulation half-life of around 23 min [173]. As a result, a few writers have advocated for the use of vehicles in ML to slowly release it and enhance the duration of action in tissues. Steady ML release using poly-lactic-co-glycolic acid micro particles has been demonstrated to convert human mesenchymal stem cells into osteoblasts. Melatonin-loaded chitosan (ML-CS) micro particles (MPs) can modulate Mel release over time, accelerating osteogenic differentiation of preosteoblast cells in vitro [86]. Local administration of 2 mg melatonin gel is a viable treatment method for effective bone and PDL regeneration in diabetic rats [174]. Melatonin has the potential to be a promising implant coating. When powdered melatonin was applied to implant sites, it caused considerably increased bone growth and bone mineralization in canines in comparison with control groups [175]. Melatonin improves the osteogenic properties of bone grafts around dental implants in canines [144]. The findings of a 3-month clinical investigation demonstrate that melatonin may be therapeutically useful in improving the Osseo integration of dental implants [176] Novel ML delivery methods, such as ML microspheres, have demonstrated tremendous potential for application in regenerative medicine and dentistry, particularly in bone-grafting techniques, to stimulate new bone growth [177].

### 6.11. Erythropoietin

Erythropoietin (EPO), a glycoprotein that is generally known as an important stimulant of erythropoiesis, is released by kidneys in adult animals and in the liver during intrauterine life [178]. Erythropoietin (EPO) is a glycoprotein hormone with a low molecular weight (30–36 kDa) that stimulates erythropoiesis. RhEPO received FDA approval in 1989, and it is now used to treat anemia caused by renal insufficiency, chemotherapy, bone marrow transplant, and AIDS [179,180,181]. EPO has non hematopoietic cellular receptors in skin, and the presence of EPO receptors on endothelial cells [150,151] and macrophages has been documented [182,183] in macrophages [184], fibroblasts, and mast cells [185,186]. Erythropoietin and its ligands are found in both the central and peripheral nervous systems [187,188]. Erythropoietin boosts anti oxidative enzyme synthesis, antagonizes glutamate cytotoxicity, influences neurotransmitter release, and induces neo angiogenesis [189]. Unlike previously held beliefs that EPO was exclusively beneficial in the formation of erythropoiesis, Epo has been shown to have multiple effects, such as tissue modulation in a variety of cell types [190,191,192,193]. There is growing evidence that EPO plays biological roles in tissues outside than the hematopoietic system, which has sparked major experimental interest. EPO is a tissue-protective hormone that promotes wound healing in a variety of damage scenarios such as tissue/organ inflammation [194]. The healing of skin lesions in rats with intentionally induced diabetes is expedited by the local administration of recombinant human EPO to the wounds, which stimulates angiogenesis, reepithelialization, and collagen deposition, while inhibiting inflammatory process and apoptosis [195]. Fibronectin supplements EPO’s positive effects on wound healing in diabetics (FN). FN promotes the establishment of the preliminary wound matrix and keeps it from dissolving [196].

Recent research has discovered that EPO also has a function in bone homeostasis. EPO may promote bone formation by boosting the production of vascular endothelial growth factor, among the most crucial factors in promoting angiogenesis and vascularization in bone repair and regeneration [197] and bone morphogenetic protein 2 [198]. Furthermore, EPO modulates bone growth via mTOR signaling [199]. According to the findings of a study conducted by Li, C. et al., EPO promotes osteoblastic activity via EphB4 signaling while increasing the amount of ephrinB2-expressing osteoclasts while reducing their resorptive actions. The combination of bidirectional signals induced by EPO via ephrinB2/EphB4 signaling resulted in bone growth [200].

Additionally, topical EPO treatment promotes palate wound healing during the early weeks following free gingival transplant surgeries [201]. A preliminary research published in 2021 by Aslroosta, H et al. demonstrated that EPO showed promise in the periodontal therapy [202]. Wang et al. discovered that erythropoietin stimulates osteogenesis and osteoinduction in a research [203]. Li, D et al. demonstrated that an injectable thermo responsive hydrogel laden with erythropoietin may successfully increase maxillary sinus floor repair in a research study led by them. [204]. It was discovered that injectable thermo sensitive hydrogels containing erythropoietin and aspirin stimulate periodontal regeneration [83].

### 6.12. Calcitonin (CTN)

Calcitonin (CTN), a hormone secreted by par follicular cells (C cells) in the thyroid gland, is crucial in bone maintenance and calcium metabolic control [205]. CTN binds to osteoclasts only in bone tissues, demonstrating the greatest expression of calcitonin receptor (CTR), and triggers osteoclast activity to cease [205,206]. CTN, according to Granholm et al., suppresses osteoclast development in mouse hematopoietic cells through modulating RANK signaling [207]. CTN has also been used to treat hypercalcemia from cancer and postmenopausal osteoporosis [208]. In rats with periodontitis, local injection of CTN reduced alveolar bone resorption through controlling osteoclast activation [87].

## 7. Limitations of Local Hormone Delivery Systems in Bone and Periodontal Tissue Engineering

Despite advances in local drug delivery systems over the last several years, the inclusion of treatments into carriers to optimally stimulate bone and periodontal regeneration remains difficult and restricts the clinical efficiency of bone and periodontal regeneration in vivo.

Natural drug carriers are biocompatible and have a minimal immune reaction.

They promote cell adhesion, proliferation, and the creation of new tissue and are ingested by biochemical breakdown [209]. However, like with any natural substance, there are drawbacks related to changes in the material’s integrity, resulting in low repeatability and restricted control over the physical characteristics [209,210,211,212]. Synthetic vehicles have a structured format, consistent material resources, extended shelf life, a low risk of toxicity, and can be produced in huge quantities with high repeatability.

Their principal drawbacks include low bioactivity, acid residues, and a monotonous architecture that provides little biological information to cells [213,214]. Despite extensive study in the sector, there are still significant limits in the use of synthetic materials as local drug carriers, mostly due to insufficient sustained release of the drugs from the scaffolds.

Although other techniques, such as the implementation of liposomes and nanoparticles, are being investigated, a quick burst release of the loaded compounds is still frequently described in research.

It implies that the outcomes of lengthier healing durations will be ineffective [215]. As a result, synthetic biomaterials may be utilized in combination with naturally occurring materials to accommodate for these drawbacks [216]. The difficulty of hormonal administration to achieve therapeutic amounts of medications at disease locations due to the hydrophobic nature of the hormone, burst release, and nonspecific absorption in healthy tissues is its fundamental drawback [215]. Furthermore, because certain compounds may be more beneficial in later phases of bone and periodontal regeneration, it would be preferable if they were delivered in a continuous and regulated manner by the biomaterials.

**Figure 6:**
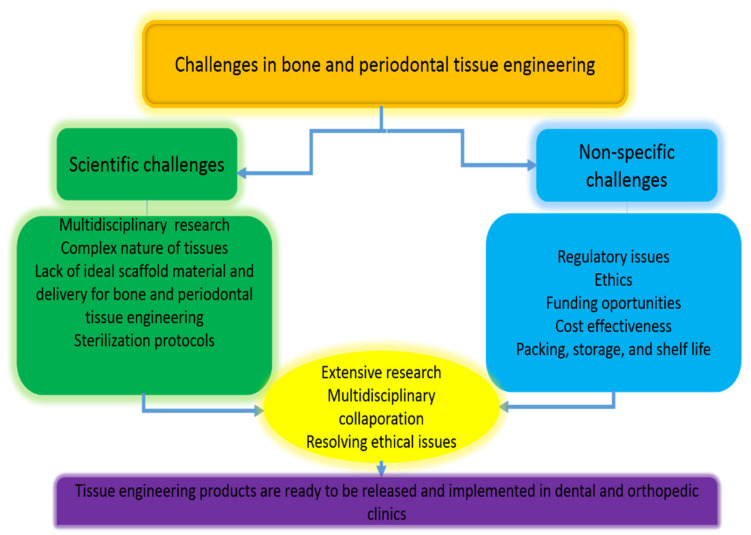
Challenges in bone and periodontal tissue engineering, reproduced and modified from Zafar et al. [217].

Furthermore, more biomaterials should be investigated in order to have a better knowledge of the impact of localized hormone administration on bone and periodontal regeneration. Figure 6 shows challenges in bone and periodontal tissue engineering.

## 8. Conclusions and Future Perspectives

As previously stated, there are new advances in recent years in improving the clinical result of oral bone and periodontal therapy. The enormous research accomplishments in tissue engineering technologies, especially in periodontium and oral bone, have empowered the research community to embrace several of the viable options for the innovation of clinically useful strategies to regenerate not only the oral bone but also the periodontium and preserve their integrity. Growth factors are very potent oral bone and periodontal growth mediators; however, they have several drawbacks, such as sophisticated, expensive processing techniques, short-half times, and poor stability. Drug repositioning regarding hormones has been considered a viable alternative for growth factors. They are better than growth factors, because hormones are much cheaper, need simple processing techniques, are more therapeutically effective, and have lower side effects. Local application of repurposed hormones shows tremendous promise for controlling processes involved in oral bone and periodontal repair. Because of the positive results obtained by these repositioned hormone delivery scaffolds, they are expected to have good therapeutic applications in the treatment of fractures, osteoporosis, periodontics, and other conditions.

However, clinical adaptations of this delivery method are currently ongoing. Nonetheless, because of the potential to promote oral bone and periodontal tissue regeneration, these delivery methods may be developed for clinical application in the near future.

## Figures and Tables

**Table 1:** Biomaterials’ benefits, drawbacks, and therapeutic applications.

Biomaterial	Advantages	Disadvantages	Clinical Application
Ceramics	Hard surface Mechanical stability Biocompatibility Osseo-conductivity	Brittleness Slow degradation Difficult processing	Bone cements Alveolar bone preservation Guided bone regeneration procedures
Natural Polymers	Biocompatibility Bioactivity	Poor mechanical properties Fast biodegradation rate	Bone tissue engineering Periodontal drug delivery Periodontal dressing
Synthetic polymers	Capability to modulate structure, porosity, and mechanical properties during fabrication.	Low biocompatibility Low mechanical strength	Sutures Bone cements Periodontal drug delivery
Composites	Biocompatibility Enhanced mechanical features	Processing difficulties	Hard and soft tissue regeneration
Hydrogels	Biocompatibility Controlled drug delivery Possibility to modulate their features during fabrication process		Hard and soft tissue regeneration

**Table 2:** The importance and difficulties of medication repurposing.

No.	Significance	Ref.	Challenges	Ref.
1	Ensures safety	[68]	Inadequate understanding of regulatory standards	[69]
2	It results in lowering tome and costs	[70]	Insufficient revenue motives	[69]
3	Opportunity for branding: increased worldwide income; drives market expansion	[65,68]	Clinical trial issues include the possibility of failed proof-of-concept studies for novel indications	[70,71]
4	Out licensing likelihood: new purposes while keeping rights to the old indication	[68]	Patent constraints obstruct the marketing of repurposed molecules	[70]
5	Satisfy unfulfilled medical needs through discovering new applications for existing medications to cure uncommon disorders and targeting tumors with non-cancer therapies	[69,72]	Economic needs assessment	[72,73]

**Table 3:** Classification of hormones.

Peptides	**Glycoproteins**	Amines	Eicosanoids	Steroid Hormones
Source: made up of amino acid residues	Source: conjugated protein bound to carbohydrate	Source: modification of amino acids	Source: small fatty acid derivatives with a variety of arachidonic acid	Source: derived from cholesterol
Short peptides e.g., Thyrotropin-releasing hormone (TRH). Intermediate peptides e.g., Insulin, and PTH	e.g., Thyrotropin (TSH)	e.g., thyroid hormones and catecholamines	e.g., Prostaglandins	Examples: Sex hormones, e.g., testosterone and estrogen Adrenal cortex hormones e.g., aldosterone, and cortisol
Short peptides		e.g., Melanocyte-stimulating hormone (MSH) Thyrotropin-releasing hormone (TRH)		
Intermediate peptides		e.g., Insulin Parathyroid hormone (PTH)		
Glycoproteins		Follicle-stimulating hormone (FSH) Thyrotropin (TSH)		
Peptide-based hormones				
Amino acid derivatives				
Iodothyronines		Thyroxin (T4) Triiodothyronine (T3)		
Amines		i.e., • Melatonin		
Steroidal hormones				
		Estrogens Testosterone (T) Cortisol Vitamin D		

**Table 4:** Current research is focuses on various hormones locally applied for bone and periodontal tissue engineering.

Hormone	Current Indication	Used Carrier	Repurposed Application	Reference
Thyroxin	Hypothyroidism and thyroid cancer	Chitosan/collagen hydrogel	Angiogenesis and neovascularization	[76]
Oxytocin	Postpartum hemorrhage, labor induction, and incomplete or inevitable abortion	Micro porous β-TCP	Osseo induction and enhanced osteogenesis	[77]
Dexamethasone	Arthritis, blood/hormone issues, allergic responses, skin illnesses, vision difficulties, respiratory problems, gastrointestinal problems, tumors, and hypersensitivity reactions are all examples of medical conditions	Chitosan-alginate-gelatin matrix	Increased proliferation and osteogenic-enhanced bone marrow	[78]
Androgens	Estradiol production, sex drive and muscular mass	PLGA-coated pericardial membranes	Enhanced implant Osseo-integration and repair of bone defects and fractures	[79]
Parathyroid Hormone	Calcium/Phosphorus homeostasis	Injectable Gelatin Methacrylate (GelMA) hydrogel	Increased ALP activity and mineralization	[80]
Insulin	Treatment of Diabetes	Poly lactic-co-glycolic-acid (PLGA) nano spheres were incorporated into nano hydroxyapatite/collagen (nHAC) scaffolds	Increased bone regeneration in rabbit mandible critical size defects	[81]
Raloxifene	Treatment and prevention of postmenopausal osteoporosis	Chitosan composite encapsulated with PLGA microspheres	Increased cell proliferation, greater mineralization capability, and ALP activity	[82]
Erythropoietin	Treatment of cancer induced anemia	Cs/β-GP/Gelatin hydrogel	Anti-inflammation and improved periodontal regeneration	[83]
Estrogen	Primary ovarian insufficiency Female hypogonadism	β-cyclodextrin/silk fibroin (SF)	Improved cell proliferation and osteoblast differentiation markers	[84]
Vitamin D	Osteomalacia, Osteoporosis	Polycaprolactone/gelatin scaffold incorporating HA nanoparticles.	Increased hADSC osteogenic development and maturation	[85]
Melatonin	Insomnia	Chitosan micro particles	Accelerating osteogenic differentiation of preosteoblast cells in vitro	[86]
Calcitonin	Hypercalcemia, Paget’s disease of bone	Local injection	Reduced alveolar bone resorption by controlling the action of osteoclasts	[87]

## Data Availability

Not applicable.
